# Preparation of magnetic ion imprinted polymer with waste beer yeast as functional monomer for Cd(ii) adsorption and detection

**DOI:** 10.1039/c9ra03859k

**Published:** 2019-07-29

**Authors:** Chunsheng Xie, Shoulian Wei, Dan Chen, Wenying Lan, Zijun Yan, Zhenxing Wang

**Affiliations:** College of Environmental and Chemical Engineering, Zhaoqing University Zhaoqing 526061 China 1430019651@qq.com +86-758-2716016; South China Institute of Environmental Sciences, Ministry of Ecology and Environment of the People's Republic of China Guangzhou 510655 China wangzhenxing@scies.org +86-15920128016

## Abstract

In this work, a magnetic ion imprinted polymer (MIIP) with specific recognition capability toward cadmium was prepared by a sol–gel method using waste beer yeast, which is a macromolecule biomass, as a functional monomer. The obtained Cd(ii)-MIIP was characterized using scanning electron microscopy (SEM), Fourier transform infrared (FTIR) spectroscopy and adsorption experiments. Then, a MIIP adsorbent based magnetic solid phase extraction (MSPE)-graphite furnace atomic absorption (GFAA) method was established to analyze the cadmium content in food and environmental samples. The maximum cadmium adsorption capacities by the MIIP and magnetic non-imprinted polymer (MNIP) were 62.74 and 32.38 mg g^−1^, respectively. The absorption by the MIIP was fitted using a pseudo-second-order kinetic model. The Cd(ii)-MIIP demonstrated superior absorption capability for selective removal cadmium. The recovery rate of the MIIP was 90.7% after four adsorption–desorption cycles. The calculated Cd(ii) detection limit (S/N = 3) was 0.18 μg L^−1^ with the relative standard deviation (RSD) equal to ∼3.5% for 10 μg L^−1^ of Cd(ii) standard solution. Our proposed method was successfully used in detecting Cd(ii) in aqueous samples. The results obtained in this work suggest that the Cd(ii)-MIIPs might be promising adsorbents to remove harmful cadmium ions from aqueous samples.

## Abstract 2

In this work, a magnetic ion imprinted polymer (MIIP) with specific recognition capability toward cadmium was prepared by a sol–gel method using waste beer yeast, which is a macromolecule biomass, as a functional monomer.

## Graphical abstract

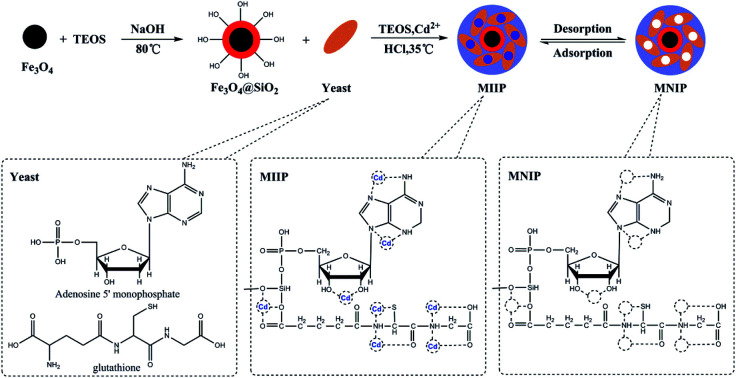

## 1. Introduction

Heavy metals as an important type of environmental contaminant should be typically removed due to toxicity. Cd(ii) is considered to be among the most toxic heavy metal environmental pollutants.^1^ The presence of Cd(ii) in food samples may occur for different reasons, such as heavy metals in natural water and/or soil or in plants that are subsequently eaten by animals.^2^ For these reasons, it is necessary to detect the Cd(ii) concentration and remove it from water and food samples. Adsorption is considered among most effective and valid method for heavy metal removal from aqueous samples. A large variety of different adsorbents were synthesized for Cd(ii) removal from natural and waste waters.^3^ Recently, the use of modified graphene^4^ and metal–organic frameworks^5^ as such an adsorbent became popular because of their high stability and surface area. Magnetic silica particles (*e.g.* Fe_3_O_4_@SiO_2_) have been attracting great attention for their magnetic features and excellent chemical and hydrothermal stability.^6,7^ Adsorbents combining with Fe_3_O_4_@SiO_2_ have also been used in magnetic solid phase extraction (MSPE) for the pre-concentration of heavy metals prior to atomic absorption spectroscopy determination.^8,9^ However, these modified nanoparticles are applied in the non-specific absorption of heavy metals and have low selectivity. Ion imprinted polymers (IIPs) possess recognition specificity designed for target ion molecules and have received widespread attention.^10^ Cd(ii) ion imprinted polymer (Cd-IIP) nanocomposites have been prepared by different methods, such as the precipitation polymerization method,^11^ sol–gel technique^12^ and combination of surface imprinting technique with sol–gel process.^13^ Magnetic ion imprinted polymers (MIIPs), in which polymers are prepared using fabricating the IIPs on the surface of a magnetic substrate (*e.g.* Fe_3_O_4_@SiO_2_), can be isolated easily from samples without tedious filtration or centrifugation during adsorption and detection.^14,15^ IIPs and MIIPs have been applied to analyzing ion matter in the fields of medicine, food contaminants, and environmental protection, and are considered as promising for heavy metal separation and/or adsorption.^14,16^ Moreover, the common applications of IIPs and MIIPs are still in solid phase extraction for the pre-concentration of heavy metals prior to determination.^10^ Recently, Cd(ii)-magnetic ion imprinted polymer (Cd(ii)-MIIP) was prepared by a surface imprinting technique combined with a sol–gel process^17^ and the surface imprinting method used dual functional monomers.^18^ Despite tremendous endeavors having been devoted to design and synthesize MIIPs for the removal of heavy metals, the report of practical applications of MIIPs in wastewater treatment is still limited.^16,19,20^ One major obstacle is that the development of cost-effective adsorbents is extremely important for practical applications in wastewater treatment.^21^ Hence, continuous improvement is needed to produce cost-effective MIIP adsorbents.

Agricultural waste and by-products are often used to remove metal ions.^21,22^ Beer brewing generates significant amount of by-products and waste, which need to be discarded safely.^23^ One of such products is waste beer yeast. It is inexpensive and shows promising adsorbent properties relative to organic compounds^24^ or heavy metal ions.^25^ The surface of waste beer yeast biomass had great amount of amide, carboxyl, and hydroxyl groups, which adsorb heavy metals. This biomass has also been modified using different chemical methods to enhance the adsorption of heavy metals.^26–28^ Recently, yeast and waste beer yeast modified with magnetic Fe_3_O_4_ nano-particles have also presented good adsorption performances and simple isolation and recycling for cationic dyes^29^ and heavy metals.^30^ However, due to the low adsorption selectivity of waste brewery biomass relative to heavy metals, improving yeast biomass adsorption selectivity by chemical treatments is important and beneficial for the application.

The molecular imprinting polymer is typically synthesized with molecular templates, functional monomers, and cross-linkers. Functional monomer is important to interact with template to form a pre-polymerization complex by providing functional groups during the preparation of MIIP. However, the number of functional monomers used in MIIP is limited and most functional monomers are small molecule compound,^10,14,18,19^ which certainly would restrict the selectivity and applicability during the application of MIIP. Previously, we demonstrated synthesis of MIIP using lead and cadmium ions as co-templates as well as acrylate-modified *S. platensis* and 4-vinylpyridine (4-VP) as dual functional monomers with the goal to use MIIP to analyze lead and cadmium contents in food and environmental samples.^31^ In this work, waste beer yeast, which is a typical macromolecule biomass, was used as a functional monomer to prepare magnetic Cd(ii)-MIIP *via* a sol–gel method to improve the specificity and selectivity of waste beer yeast. Moreover, using waste beer yeast as a functional monomer to produce low-cost and high adsorption capacity magnetic nanocomposite, which was then implemented in pre-concentration and separation of cadmium ions in a MSPE-GFAA method for cadmium analysis in environmental and food samples. MIIP was characterized using Fourier-transform infrared spectroscopy (FTIR) and scanning electron microscopy (SEM). Adsorption performance, selectivity, reusability and application of this new MIIP for cadmium determination were thoroughly investigated and discussed.

## 2. Materials and methods

### 2.1 Chemicals and materials

Cd(NO_3_)_2_, Pb(NO_3_)_2_, Mg(NO_3_)_2_, Zn(NO_3_)_2_, MnSO_4_, Ni(NO_3_)_2_ and Cu(NO_3_)_2_ were supplied by Aladdin Reagent Company (Shanghai, China). Ferric and ferrous chlorides (FeCl_3_·6H_2_O and FeCl_2_·4H_2_O, respectively) were obtained from Tianjin Chemical Reagent Co. (China). Ethylenediamine tetra-acetic acid (EDTA, AR), ammonium ferric sulfate (NH_4_FeSO_4_·H_2_O, AR), ammonia (NH_3_·H_2_O, AR), sodium hydroxide (NaOH, AR), methanol and ethanol were purchased from Guangzhou Chemical Reagent Factory (GCRF). Tetraethyl orthosilicate (TEOS) was obtained from Shanghai Chemical Reagent Factory. The waste beer yeast was from Zhaoqing Pabst Blue Ribbon Co.

### 2.2 Instrumentation

Graphite furnace atomic absorption (AA-7000, Shimadzu, Japan) was implemented for cadmium concentration determination. FTIR spectra were obtained using FTIR-8400S infrared spectrophotometer (Shimadzu, Japan). A Philips XL-30 scanning electron microscope was using to observe the SEM images. The polymer was placed under vacuum in an ADP310C model vacuum drying oven (Yamato, Japan). A super magnet with a magnetic field of 0.35 Tesla (10 × 5 × 1 cm) was used to magnetic separation.

### 2.3 Preparation of Fe_3_O_4_@SiO_2_

Fe_3_O_4_@SiO_2_ was synthesized by chemical co-precipitation according to previously reported method^31^ with some modifications. To synthesize Fe_3_O_4_ nanoparticles, 11.2 and 5.6 mmol L^−1^ of ferric and ferrous iron chlorides, respectively, were dissolved in 150 mL of distilled water. The solution was degassed with nitrogen for 15 min to remove oxygen and heated to 80 °C, after which NH_3_·H_2_O was added to the mixture under constant stirring at 500 rpm. The whole system was held at 80 °C for another 30 min. Fe_3_O_4_ were collected by a magnet. The supernatant was discarded. The adsorbent was rinsed 3–4 times with ethanol and distilled water until pH was 7.0 to remove impurities. The supernatant was dried in a vacuum and weighed. The resulting Fe_3_O_4_ nanoparticles (∼160 mg) were dispersed in a mixture containing 3.5 mL of 2 M NaOH and 480 mL of water and stirred for 15 min at 80 °C. 2 mL of TEOS was added gradually over 5 minutes. The ratio of the substances in the reaction was SiO_2_ : H_2_O = 1 : 70. Reaction time was 2 hours, after which the composite was rinsed 3–4 times with ethanol and dried at 120 °C for 1 hour.

### 2.4 Fe_3_O_4_@SiO_2_@IIP preparation

**Fig. 1:**
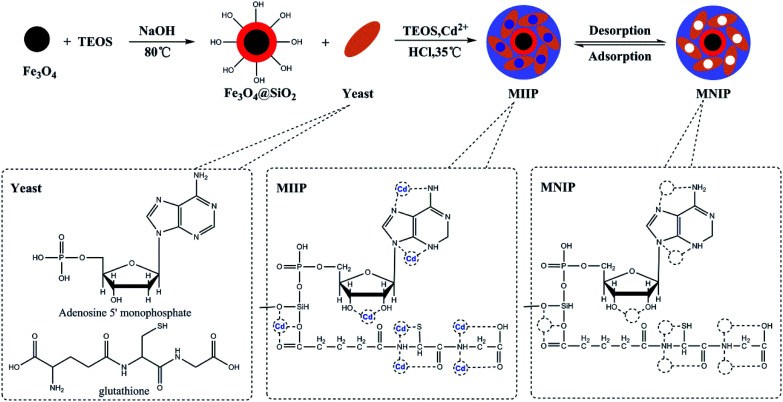
Stages of preparation of the Cd(ii)-MIIP.

The waste beer yeast sample was cultured and pretreated for ethyl orthosilicate modification. The mycelium was separated using a 180 μm sieve. The sieved products were ground and sterilized, washed with distilled water and centrifuged at 4000 rpm for 30 min. The resulting beer yeast was stored at 4 °C until the next step. The preparation of the Fe_3_O_4_@SiO_2_@IIP was carried out using a sol–gel surface imprinting method. 1.0 g of Fe_3_O_4_@SiO_2_ was mixed with 50 mL of methanol for 20 min. In a separate container, 5.0 g of dry beer yeast, 5 mL of TEOS and 5 mL of 2 mol L^−1^ HCL and 1.0 mmol L^−1^ Cd(NO_3_)_2_ were mixed with 150 mL of distilled water and kept at 35 °C in a water bath for 1 h, after which the solution was mixed with the first solution containing Fe_3_O_4_@SiO_2_ and 1.0 mmol L^–1^ Cd(NO_3_)_2_ and stirred for 24 h. At the end of the reaction, Fe_3_O_4_@SiO_2_@IIP was collected by a magnet, rinsed with 0.5 mol L^−1^ EDTA solution under ultrasonication until Cd(ii) can not to be detected. The obtained MIIP were collected, washed with deionized water for three times and then freeze-dried. Magnetic non-imprinted polymer (MNIP) was prepared using the same procedure (but without Cd(NO_3_)_2_ template) as a reference. Schematics of this synthesis procedure are shown in Fig. 1.

### 2.5 Adsorption experiments

Adsorption experiments were conducted using a batch method.^31^ 30.0 mg of MIIP (or MNIP, waste beer yeast and Fe_3_O_4_@SiO_2_) was mixed with 100 mL of solution containing Cd(ii). pH of this solution was adjusted to 6.0 using sodium acetate and acetic acid solutions. The solutions were stirred at 150 rpm for 2 h at 25 °C. The resulting MIIP (or MNIP, waste beer yeast and Fe_3_O_4_@SiO_2_) was collected by a magnet. The supernatant was analyzed by GFAA.

For dynamic adsorption, 30 mg of dry MMIP or MNIP was stirred into 10.0 mL of 1.0 mg mL^−1^ cadmium solution with pH = 6.0 at 25 °C. Cadmium content in the solution was analyzed at different time points, allowing the adsorption quantity (*Q*) to be calculated at different times; the adsorption kinetics curve was then drawn.

### 2.6 Selectivity experiments

To test selectivity, absorption experiments were conducted with other divalent metal ions as structural analogues of the Cd(ii) ions as well as with Mg(ii) as a reference metal ion.^31^ For these tests, 100.0 mg of the sorbent (MIIP or MNIP) was added to 25.0 mL of a standard solution containing 1.0 × 10^−3^ mol L^−1^ of either Cu(ii), Mg(ii), Zn(ii), Mn(ii) or Ni(ii) at pH = 6.0. The solution was shaken and allowed to adsorb for 1 hour at 25 °C, and then the supernatant was analyzed using graphite furnace atomic absorption. The selectivity coefficient and imprinting factor were obtained from the formulas below^20^

1 *α* = *Q*_MIIP_/*Q*_MNIP_

2 *β* = *α*_X_/*α*_Mg(II)_

where *α* is an imprinting factor, *Q*_MIIP_ and *Q*_MNIP_ are adsorption concentrations of Cd(ii), Pb(ii), Cu(ii), Mg(ii), Zn(ii), Mn(ii), and Ni(ii) on MIIP and MNIP, respectively, (in mg g^−1^), *β* is selectivity coefficient, *α*_X_ is an imprinting factor for other ions and *α*_Mg(II)_ is the imprinting factor of Mg(ii).

### 2.7 MSPE procedure and Cd(ii) detection

In a 100 mL beaker, 0.03 g MIIP, 10.0 mL of the sample solution, a suitable amount of HCl or NaOH to change pH of the solution to 6.0 were combined. Ultrasonic treatment was applied at room temperature for activation, and the mixture underwent ultrasonication for 10 minutes, followed by magnetic separation, washing twice, rinsing twice with 10.0 mL deionized water, and magnetic separation. The eluent was rinsed with 10.0 mL of 0.5 mol L^−1^ EDTA solution for 10 minutes, and the solution was evaporated using a flow of nitrogen. Concentrate volume was adjusted to 50 mL by 0.1% nitric acid. The concentration of Cd(ii) was analyzed using a GFAA method.

### 2.8 Sample preparation

Blue Ribbon beer (originating from Hebei Tangshan A20130610) and Qingdao beer (originating from Shandong Qingdao A20130514) were obtained from a commercial market for the detection experiments. The CO_2_ was removed from the beer samples by shaking for 10 min. 10.0 mL beer samples were added to 4.5 mL nitric acid for microwave digestion. The solution was diluted to 100 mL with double distilled water for detection. All experiments were performed twice. The final value was an average of the two values obtained from these two different experiments.

## 3. Results and discussion

### 3.1 Characteristics of the MIIP and MNIP

#### 3.1.1 SEM analysis

**Fig. 2:**
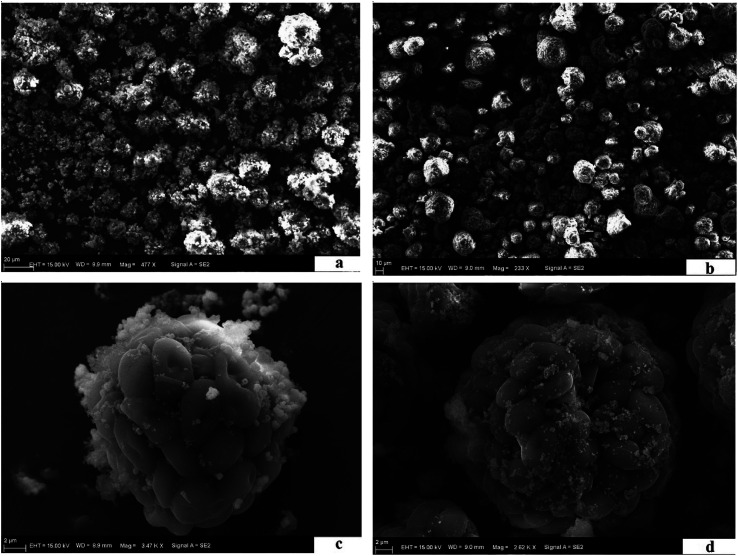
SEM of MIIP (a and c) and MNIP (b and d).

The morphological features of the MIIP (a and c) and MNIP (b and d) were analyzed by SEM (see Fig. 2). Fig. 2a and b show that both MIIP and MNIP were round and had good dispersivity and regular particle size. Average particle sizes of MIIP and MNIP were ∼20 μm. Fig. 2c and d show in both MIIP and MNIP, the yeast cells were oval, and many yeast cells accumulated on the round particles. Fig. 2c and d also show that the surfaces of MIIP and MNIP appeared to be smooth and even; however, the biomass clung together. This can be attributed to the small, round MIIP obtained by sol–gel procedure, which led the waste beer yeast to agglomerate. Morphological features of MIIP fabricated by sol–gel method using waste beer yeast as the functional monomer were obviously different from traditional baking yeast modified by nano-Fe_3_O_4_ using glutaraldehyde cross-linking agent.^32^ In this study, there were no significant differences were observed when MIIP or MNIP were used. Different adsorption capacities of MIIP and MNIP were due to the imprinting effect and not due to their morphological differences.^31^

#### 3.1.2 FTIR analysis

**Fig. 3:**
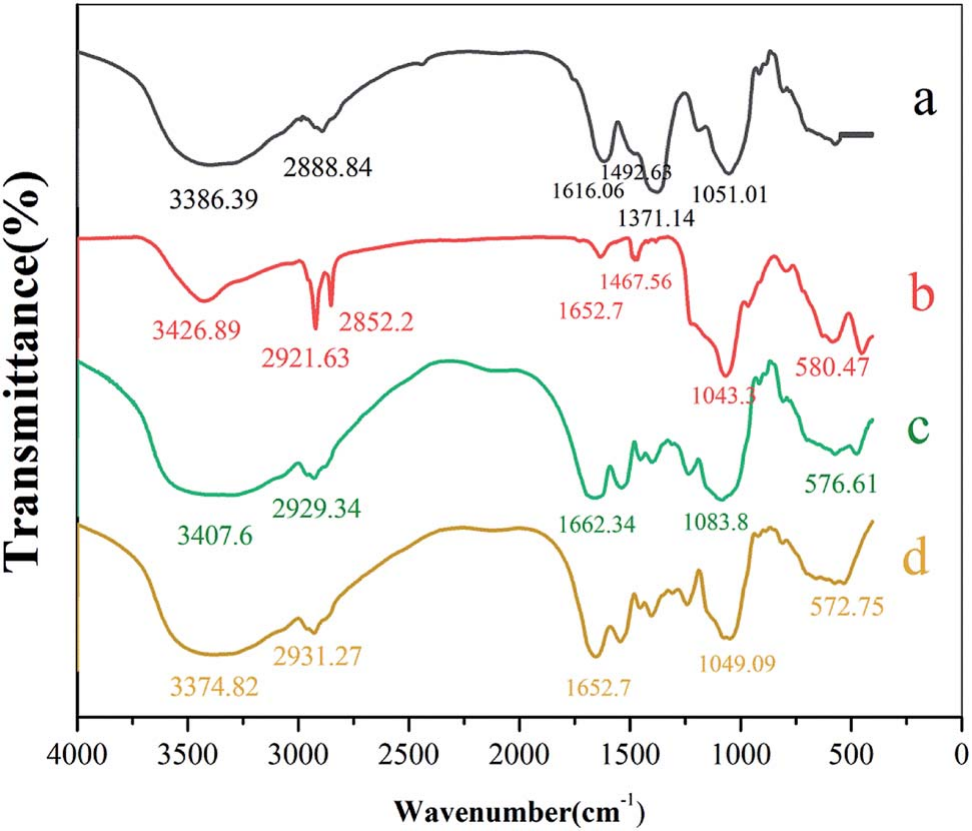
FTIR spectra of (a) waste beer yeast, (b) Fe_3_O_4_@SiO_2_, (c) MIIP and (d) MNIP.

FTIR spectra of waste beer yeast, Fe_3_O_4_@SiO_2_, MIIP and MNIP are shown in Fig. 3. Waste beer yeast spectrum had a complex structure with numerous absorption peaks (see Fig. 3a).^30^ The absorption bands of the yeast infrared spectrum were mainly composed of the characteristic peaks of proteins, carbohydrates, and hydroxyl groups. The wide and strong band at 3386.89 cm^−1^ corresponds to –OH, –NH, and –SH stretching from glutathione and adenosine 5′ monophosphate, *etc.*, in the waste beer yeast.^30,33^ Peak at 2888.84 cm^−1^ corresponds to the –CH_2_ and –CH_3_ asymmetric stretching vibrations. Peak at 1051.01 cm^−1^ was attributed to absorption of C–O–C from the RNA and DNA in the yeast or the C–O stretching from carbohydrates or alcohols in the yeast cell walls.^34^ Peak at 1616.06 cm^−1^ corresponds to C=O in benzene or to the stretching vibrations of –COOH and –NH_2_. Peak at 1371.14 cm^−1^ represented the –CH_3_ bending vibration, and the C–N and C–S stretching vibrations from the amide II band. Peak at 580.47 cm^−1^ corresponds to absorption of Fe–O (see Fig. 3b). Strong peak at 1043.3 cm^−1^ corresponds to characteristic absorption peak of Si–O–Si. Thus, SiO_2_ was successfully applied to the Fe_3_O_4_ surface.^35^ FTIR spectrum of beer yeast is very different from spectra of MIIP and MNIP (see Fig. 3). Characteristic absorbance peaks of yeast (from 1616.06 to 1051.01 cm^−1^) exhibited a great offset in the MIIP and MNIP spectra (curve c and d), which was attributed to their combination with Fe_3_O_4_@SiO_2_ through the condensation reaction. The characteristic absorption peak of Fe–O appeared at about 576.61 cm^−1^ (c) and 572.75 cm^−1^ (d) for MNIP and MIIP, respectively, indicating successful encapsulation of MIIP onto the waste beer yeast.^31,33^ FTIR spectra of MIIP and MNIP are similar: MIIP bands are just slightly offset from those of MNIP. These results showed that the magnetic ion imprinted polymer were successfully synthesized.

### 3.2 Adsorption behavior of MIIP

#### 3.2.1 Static adsorption

**Fig. 4:**
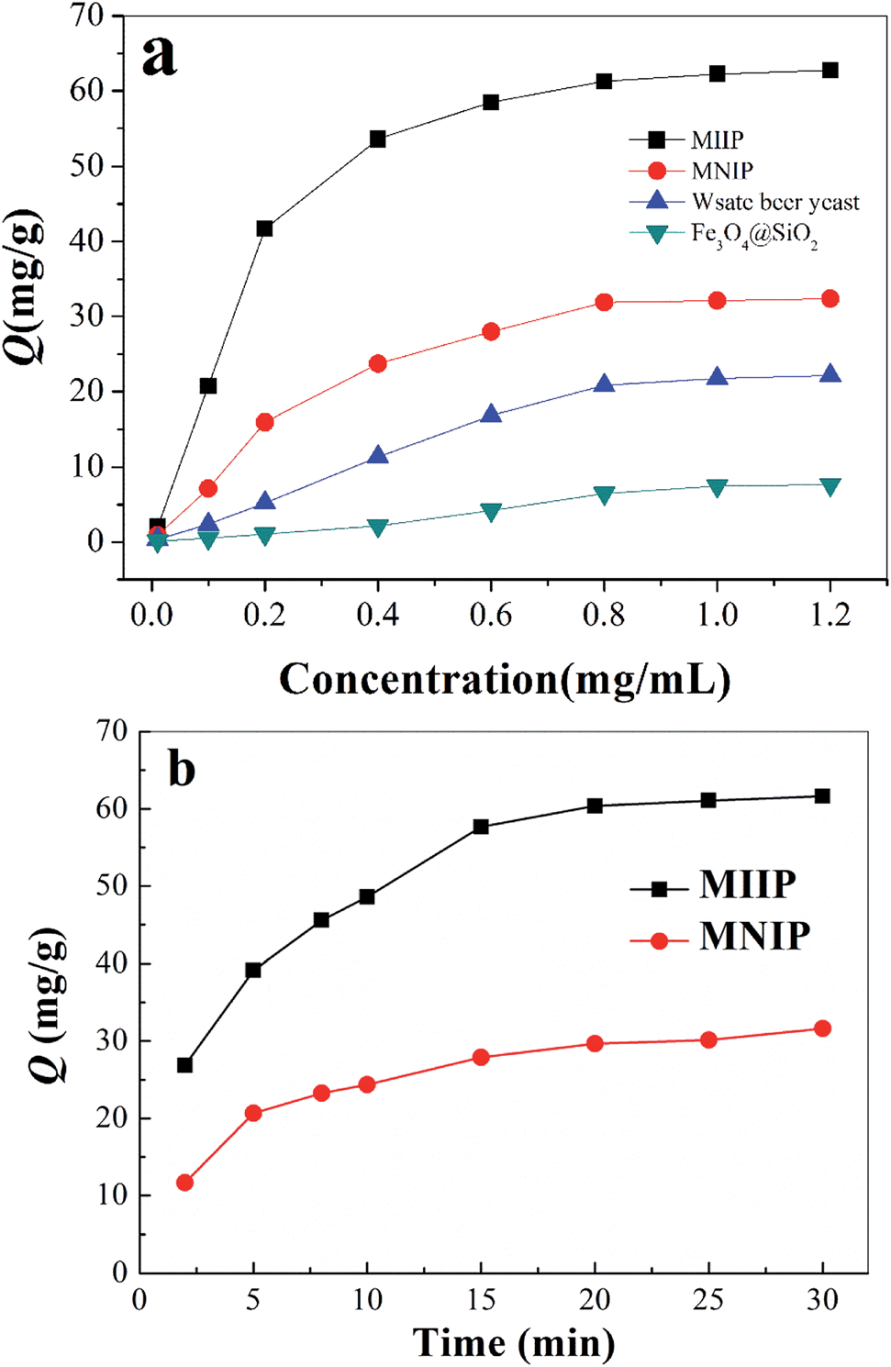
Adsorption behavior of MIIP: (a) static adsorption; (b) dynamic adsorption.

Fig. 4a shows adsorption isotherms of MIIP, MNIP, waste beer yeast and Fe_3_O_4_@SiO_2_ relative to cadmium. Adsorption capacity of MIIP, MNIP, waste beer yeast and Fe_3_O_4_@SiO_2_ increased as cadmium concentration increased. At cadmium concentration of ∼1.0 mg mL^−1^, adsorption capacities of MIIP and MNIP were mostly unchanged, indicating that the adsorption was saturated. Heavy metal adsorption using different types of yeasts has been studied by various investigators. The respective adsorption capacities of different yeast biomasses are presented in Table 1 for comparison with the studied modification methods. Maximum adsorption capacities of MIIP and MNIP relative to Cd(ii) were 62.74 and 32.38 mg g^−1^, respectively. Magnetically modified biomass significantly higher adsorption capacity towards Cd(ii). Maximum adsorption capacity of MIIP and MNIP towards cadmium reported in this work are higher than those reported for Cd(ii) ion imprinted polymer prepared by a sol–gel method.^12,13^ Maximum adsorption capacity of MIIP was two times higher than that of MNIP because of specific binding sites. Cadmium adsorption by MNIP was only non-specific. The adsorption capacities of waste beer yeas and Fe_3_O_4_@SiO_2_ relative to Cd(ii) were 22.15 and 7.62 mg g^−1^, respectively, which are significantly lower than MIIP and MNIP.

**Table 1:** Heavy metal adsorption capacities of yeast biomass using different modification methods

Heavy metal	*Q*_max_ (mg g^−1^)	Yeast	Modification method	Ref.
Pb(ii)	5.72	Waste beer yeast	None	25
Cu(ii)	1.45	Waste beer yeast	None	25
Pb(ii)	20.0	Baker's yeast	None	28
Cu(ii)	4.5	Baker's yeast	None	28
Cd(ii)	57.29	Dead yeast cells	None	36
Cd(ii)	110	Native baker's yeast	None	37
Cd(ii)	15.63	Waste baker's yeast	Ethanol	38
Cd(ii)	45.87	Baker's yeast	Cystine	39
Cd(ii)	41.55	Baker's yeast	EDTA dianhydride and magnetic Fe_3_O_4_	30
Cd(ii)	122.10	Dead yeast cells	Immobilized in Na-alginate	36
Cd(ii)	102.80	Baker's yeast	Crosslinked with *β*-cyclodextrin polymers	40
Cd(ii)	62.74	Waste beer yeast	Magnetic ion imprinted polymer (MIIP)	This work
Cd(ii)	32.38	Waste beer yeast	Magnetic non-imprinted polymer (MNIP)	This work

#### 3.2.2 Dynamic adsorption

Dynamic adsorption results are shown in Fig. 4b. Cadmium adsorption rate by MIIP and MNIP was fairly fast at the beginning; the adsorption saturation of MIIP for Cd(ii) was reached in 20 minutes. It was possible that MIIP easily reached the saturation state due to the sol–gel surface imprinting. Comparison of MIIP and MNIP adsorption curves demonstrated greater cadmium adsorption by MIIP than by MNIP at the same adsorption time.

Cadmium equilibrium amounts adsorbed by MIIP and MNIP were 62.74 and 32.38 mg g^−1^, respectively. Thus, MIIP demonstrated high adsorption capacity as well as a good imprinting effect. The pseudo-first-order and pseudo-second-order models (shown by eqn (3) and (4), respectively) were applied separately to understand the kinetics of the adsorption mechanism:^41,42^3
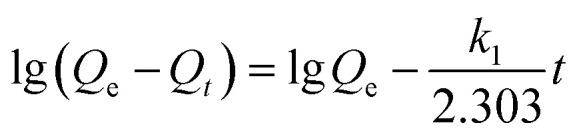
4
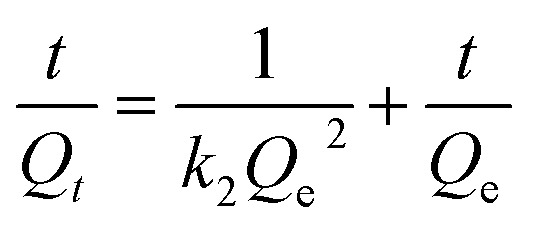
where *Q*_e_ is cadmium content adsorbed by MIIP and MNIP at equilibrium (in mg g^−1^) and *Q*_*t*_ is time *t* (in min); *k*_1_ (in min^−1^) and *k*_2_ (in g mmol^−1^ min^−1^) are the pseudo-first- and pseudo-second-order adsorption rate constants, respectively. Theoretical value *Q* estimated from the pseudo-second-order model was closer to the experimentally-obtained value. This model fitted the adsorption data better than the pseudo-first-order one (see Table 2). Thus, chemical interactions, such as hydrogen bonding between the sorbent and the adsorbate, very likely control adsorption processes, and that the adsorption rate is dominated by Cd(ii) diffusion in the solution. In the case of the adsorption by the imprinted polymer, adsorbed ions first reach the outer surface of the microparticles by diffusion, which is external diffusion. The ions then pass through the surface pores of the particles into their inner surface, which is internal diffusion.^35^ Therefore, initial Cd(ii) adsorption stage by the particles is mainly based on physical adsorption, which satisfies the first-order adsorption kinetic model better. As physical adsorption progresses, functional groups of the adsorbed ions and the inner and outer surfaces of the particles form covalent or ionic bonds, and chemical adsorption occurs to control the adsorption rate of the whole process. Therefore, pseudo-secondary adsorption kinetic model can better satisfy the overall adsorption process and agrees with experimental results.

**Table 2:** Kinetics constants for the adsorption of Cd(ii) by MIIP and MNIP

Sorbent	*Q*_e,exp._ (mg g^−1^)	Pseudo first order kinetic model			Pseudo second order kinetic model		
		*Q*_e,cal._ (mg g^−1^)	*k*_1_ (min^−1^)	*R* ^2^	*Q*_e,cal._ (mg g^−1^)	*k*_2_ (min^−1^)	*R* ^2^
MIIP	62.74	44.53	0.1294	0.9790	69.93	0.0038	0.9974
MNIP	32.38	22.62	0.1050	0.9754	35.59	0.0068	0.9988

### 3.3 Selective behavior of MIIP

Table 3 shows adsorption capacity (*Q*), imprinting factor (*α*), and selectivity coefficient (*β*) of Cd(ii), Pb(ii), Cu(ii), Zn(ii), Mn(ii), Ni(ii), and Mg(ii) for MIIP and MINP. It can be found that MIIP had larger adsorption capacity (*Q*) for metal ions than MNIP. MIIP adsorption capacity (*Q*) towards Cd(ii) and Pb(ii) was much higher than for other metal ions because of their high molecular weight. At the same time, the imprinting factor (*α*) value of the Cd(ii)-MIIP (2.93) was nearly three times larger than that of Cd(ii)-MNIP, revealing the high selectivity of MIIP toward Cd(ii) adsorption. Moreover, the selectivity coefficient (*β*) value of the Cd(ii) (2.32), more than two times greater than 1, also shows a much higher binding specificity of MIIP for Cd(ii) ions than Pb(ii) and other metal ions. As shown in Table 3, comparison of the imprinting factor (*α*) and selectivity coefficient (*β*) for these ions showed that the MIIP competitive metal ion affinity can be expressed as the following order: Cd(ii) > Cu(ii) > Pb(ii) > Zn(ii) > Ni(ii) > Mn(ii) > Mg(ii). Thus, MIIP demonstrated strong adsorptive selectivity towards cadmium ions from the solution containing other metal ions. This is because the MIIP had a Cd(ii)-specific recognition cavity on its surface, which could adsorb the imprinted ion Cd(ii) specifically.

**Table 3:** Results of adsorption selectivity

Ion	*Q*/(mg g^−1^)		*α*	*β*
	MIIP	MNIP		
Cd(ii)	59.22	20.23	2.93	2.32
Cu(ii)	17.53	8.90	1.97	1.56
Pb(ii)	60.37	31.08	1.94	1.54
Zn(ii)	17.81	9.81	1.81	1.44
Ni(ii)	16.56	9.39	1.76	1.40
Mn(ii)	15.44	9.33	1.65	1.31
Mg(ii)	3.67	2.92	1.26	1

### 3.4 Optimization of the conditions for MSPE

#### 3.4.1 Adsorption conditions

**Fig. 5:**
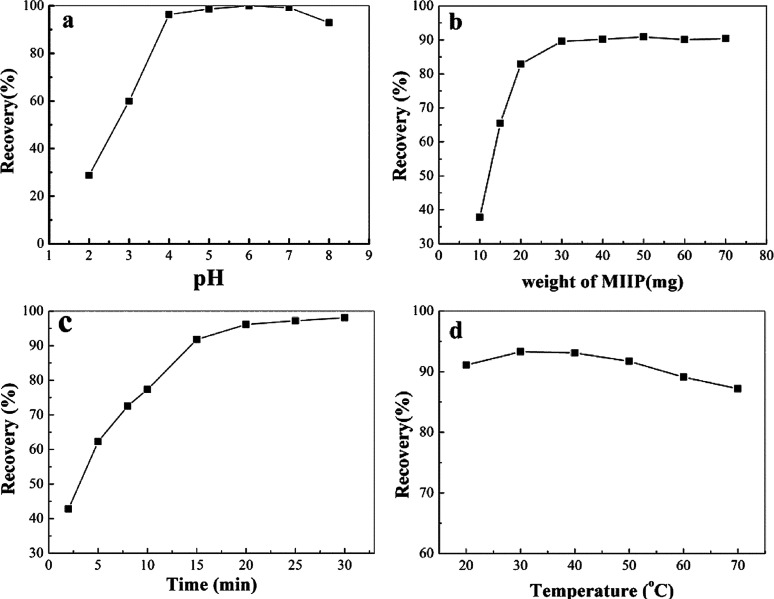
Recovery rate experiments: (a) pH; (b) dosage of MIIP; (c) adsorption time; (d) temperature.

The effect of the pH (Fig. 5a), dosage (Fig. 5b), time (Fig. 5c), and temperature (Fig. 5d) on the recovery rate by the MIIP was investigated. As shown in Fig. 5a, pH range that influenced recovery rate of MIIP towards cadmium was 2.0–8.0. At pH values below 4.0, cadmium adsorption by MIIP decreased significantly. At pH = 4.0–7.0, recovery rate reached a maximum, and then decreased slowly with increasing pH. At pH above 6.0, some cadmium was bound by formation of solid Cd(OH)_2_.^43^ Therefore, we considered pH value equal to 6.0 as the most favorable for the following absorption experiments. Influence of MIIP dosage on adsorption recovery under the same conditions is shown in Fig. 5b. With the increase of the MIIP dosage, the recovery rate is increasing. When the MIIP dosage is more than 30 mg, the recovery rate reaches a stable point. Therefore, the optimum amount of MIIP was 30 mg. Fig. 5c shows the effect of different adsorption times (2–30 min) on the recovery rate under the same conditions. Thus, recovery rate increased with time reaching its highest level at 20 min. Thus, for comprehensive efficiency and practical applications, adsorption time equal to 20 min was chosen. Fig. 5d shows temperature dependence of the MIIP adsorption capacity towards cadmium: it increased from 20 to 30 °C and decreased at temperatures above 30 °C. This experiment showed that 30 °C was the best temperature for cadmium adsorption by MIIP. The main reason is that the kinetic and thermodynamic equilibria were based on ion adsorption. It is not easy to reach the adsorption equilibrium when the temperature is too low, but it is difficult for Cd(ii) to be adsorbed by MIIP because of the intense molecular thermal movement at high temperature.

#### 3.4.2 Elution conditions

**Fig. 6:**
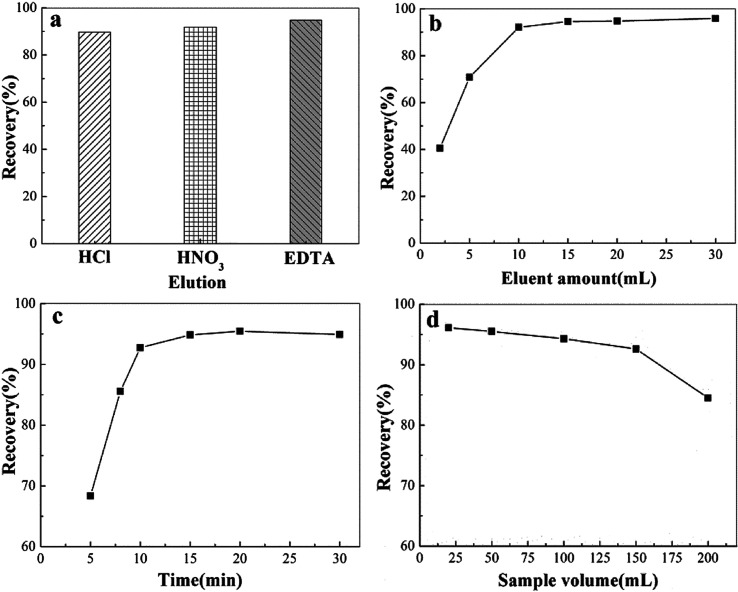
Elution condition experiments: (a) elution type; (b) eluent amount; (c) time; (d) sample volume.

In order to select the best eluent, we used HCl, HNO_3_ and EDTA (all with concentration of 0.5 mol L^−1^) to investigate the elution effect. As shown in Fig. 6a, EDTA was the best eluent, which for its highest recovery rate owing to its strong ability to sequester metal ions. Then, the effect of the volume of 0.5 mol L^−1^ EDTA on the extraction efficiency was investigated to select the best sorbent dosage. As shown in Fig. 6b, the maximum extraction recovery was achieved when the eluent volume reached 10.0 mL. Therefore, 10.0 mL was selected as the best eluent volume. Elution time is a key factor in MSPE. The effect of different ultrasonication times (0–30 min) on the elution efficiency was investigated. It was found that 10 min was enough to achieve a high recovery rate (Fig. 6c). The recovery rate in the 20–30 min range had a slight downward trend. It is possible that the eluted Cd(ii) was re-adsorbed by MIIP when the elution time was longer than 20 minutes. Therefore, taking efficiency into account, the best elution time is 10 min. In practical application, the sample volume is an important factor in adsorption. Thus, we studied how different sample volumes affect adsorption recovery. The ideal sample volume is 150 mL, as shown in Fig. 6d. As shown in Fig. 6d, the recovery rate was over 95% when the sample volume was in the range of 25–150 mL. When the sample volume exceeded 150 mL, the recovery rate decreased. It is because that the adsorbent could not contact the template well due to excessive sample volume. The ideal sample volume is 150 mL.

### 3.5 Repeatability and stability of MIIP

**Fig. 7:**
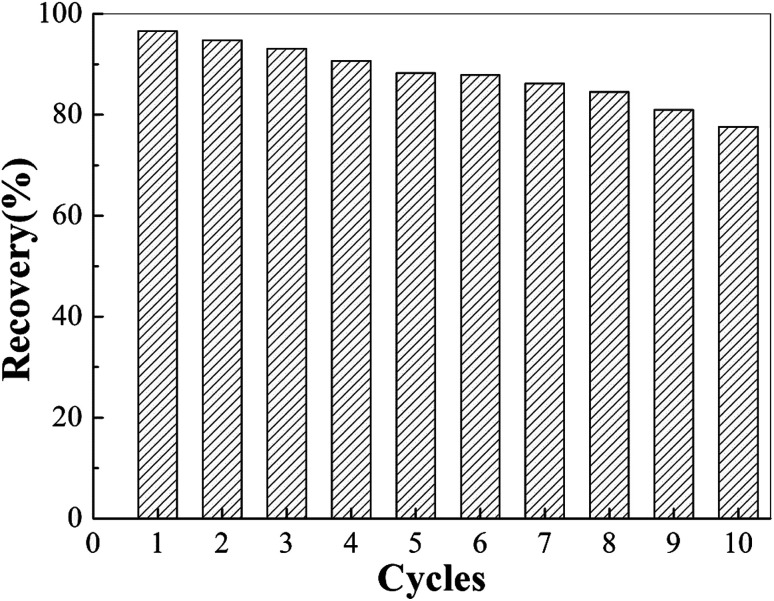
Adsorption capacity as function of number of adsorption/desorption cycles.

To investigate the stability and reusability of the MIIP as a sorbent, we performed 10 consecutive adsorption/desorption cycles with the same MIIP sorbent. The results showed that the recovery rate of MIIP was 90.7% after four adsorption–desorption cycles, 81.0% after eight adsorption–desorption cycles, and 73.6% after ten adsorption–desorption cycles (Fig. 7). This may be due to the slight swelling of the adsorbent and/or a small amount of the adsorbed heavy metals not being eluted completely. Thus, MIIP demonstrated good stability and reusability as adsorbent for cadmium.

### 3.6 Establishment and application of the MSPE-GFAA method

MSPE-GFAA method under the optimized conditions was used to determine cadmium content in the samples. Correlation coefficients of the two standard curves were both greater than 0.999, indicating that the linearity was excellent. The linear range was 0.25–5.0 μg L^−1^ (*Y* = 0.00145 + 0.0177*X*, *R*^2^ = 0.99901). The Cd(ii) detection limit (S/N = 3) was calculated to be 0.18 μg L^−1^ after 10 repeated measurements of the blank solution under optimized conditions. Relative standard deviation (RSD) of 10.0 μg L^−1^ Cd(ii) standard solution was found to be 3.5% based on 10 repetitions.

Using the optimized experimental conditions, established magnetic solid phase extraction (MSPE)-graphite furnace atomic absorption (GFAA) method was used to determine cadmium content in food and environmental samples (see Table 4). Cadmium recovery in the samples ranged from 83.6 to 96.4% with relative standard deviation below 3.9%. These results showed that the method was accurate and reliable. The measured values in water samples and beer were at a normal level.

**Table 4:** Cadmium determination in various samples

Samples	Found (μg L^−1^)	Added (μg L^−1^)	Recovery (%)	RSD (%)
Drinking water	0.15	0.5	92.2	2.2
		2.0	89.6	0.6
		4.0	87.6	1.9
Mountain spring water	0.10	0.5	83.6	3.9
		2.0	92.5	1.5
		4.0	96.4	2.6
Blue Ribbon beer	0.16	0.5	88.2	1.3
		2.0	94.9	2.7
		4.0	91.4	1.2
Qingdao beer	0.16	0.5	91.6	1.2
		2.0	92.5	0.7
		4.0	93.1	1.1

## 4. Conclusions

We successfully prepared a novel magnetic porous ion-imprinted polymer by sol–gel method using waste beer yeast as a functional monomer and Cd(ii) as a template. The resulting Cd(ii)-MIIP composites demonstrated high selectivity, fast adsorption rate, large adsorption capacity and good reusability and stability towards cadmium ions. Cadmium absorption by MIIP could be well-fitted using pseudo-second-order kinetic model. Comparing to MNIP, MIIP showed stronger ability to selectively adsorb cadmium ions from the solution containing mixed metal ions. Cd(ii)-MIIP was successfully used for rapid separation and enrichment of cadmium ions in food and environmental samples for further analysis. Accuracy and precision of our method were satisfactory. Hence, MIIP prepared by the sol–gel technique using waste beer yeast as a functional monomer is promising, low cost, effective, selective and recyclable adsorbent for heavy metals.

## Conflicts of interest

There are no conflicts to declare.

## Supplementary Material
